# Supplementary material for: Dynamic cortical and tractography atlases of proactive and reactive alpha and high-gamma activities
Source: Brain Commun. 2023 Apr 4;5(2):fcad111. doi: 10.1093/braincomms/fcad111 (PMC10204271; doi:10.1093/braincomms/fcad111)
Supplement: fcad111_Supplementary_Data [file fcad111_Supplementary_Data.docx]

**Supplementary document**

**in**

**Dynamic cortical and tractography atlases of proactive and reactive alpha and high-gamma activities**

Hiroya Ono ; Masaki Sonoda ; Kazuki Sakakura ; Yu Kitazawa ;

Takumi Mitsuhashi ; Ethan Firestone ; Jeong-Won Jeong; Aimee F. Luat ;

Neena I. Marupudi ; Sandeep Sood ; Eishi Asano.

This document includes **Supplementary Table 1.**

**Supplementary Table 1: The number of electrodes at given regions of interest (ROIs).**

| ROIs | Left | Right | Total |
| --- | --- | --- | --- |
| frontal eye field (FEF) | 23 | 30 | 53 |
| anterior fusiform gyrus (aFG) | 10 | 23 | 33 |
| posterior fusiform gyrus (pFG) | 36 | 36 | 72 |
| anterior inferior-frontal gyrus (aIFG) | 22 | 36 | 58 |
| posterior inferior-frontal gyrus (pIFG) | 78 | 82 | 160 |
| inferior parietal lobule (IPL) | 18 | 40 | 58 |
| anterior inferior-temporal gyrus (aITG) | 15 | 22 | 37 |
| posterior inferior-temporal gyrus (pITG) | 23 | 16 | 39 |
| lateral-occipital gyrus (LOG) | 65 | 67 | 132 |
| anterior middle-frontal gyrus (aMFG) | 52 | 96 | 148 |
| posterior middle frontal gyrus (pMFG) | 55 | 81 | 136 |
| anterior middle-temporal gyrus (aMTG) | 22 | 19 | 41 |
| posterior middle-temporal gyrus (pMTG) | 53 | 38 | 91 |
| paracentral gyrus (PCL) | 7 | 11 | 18 |
| postcentral gyrus (PoCG) | 103 | 105 | 208 |
| parahippocampal gyrus (PHG) | 4 | 10 | 14 |
| posterior cingulate gyrus (pCG) | 15 | 22 | 37 |
| precentral gyrus (PreCG) | 72 | 125 | 197 |
| precuneus (PCun) | 6 | 18 | 24 |
| anterior striatal gyrus (aSG) | 34 | 54 | 88 |
| posterior striatal gyrus (pSG) | 20 | 16 | 36 |
| superior frontal gyrus (SFG) | 37 | 71 | 108 |
| superior parietal lobule (SPL) | 16 | 12 | 28 |
| anterior superior-temporal gyrus (aSTG) | 27 | 40 | 67 |
| posterior superior-temporal gyrus (pSTG) | 55 | 59 | 114 |
| supramarginal gyrus (SMG) | 81 | 73 | 154 |
| medial orbitofrontal gyrus (MOrb) | 2 | 1 | 3 |
| temporal pole (TP) | 0 | 2 | 2 |
| entorhinal gyrus (Ent) | 1 | 4 | 5 |
| anterior cingulate gyrus (aCG) | 1 | 5 | 6 |

**Supplementary Table 1. The number of electrodes at regions of interest (ROIs).** The ROI analysis was not performed at the medial orbitofrontal gyrus (MOrb), temporal pole (TP), entorhinal gyrus (Ent), or anterior cingulate gyrus (aCG) because of the limited number of electrode sites eligible for analysis.
